# Multiscale Characterization and Evaluation of Low-Energy Bird-Strike Damage in CFRP

**DOI:** 10.3390/ma19091830

**Published:** 2026-04-29

**Authors:** Hongshuai Huang, Bowen Yang, Yu Cao, Zhongxian Tan, Junchao Li, Shaolong Li, Tian Tan, Wenfeng Yang

**Affiliations:** 1 College of Aviation Engineering, Civil Aviation Flight University of China, Guanghan 618307, China; click010828@163.com (H.H.); 13882665957@163.com (B.Y.); zhongxiantan123@163.com (Z.T.); 13551679056@163.com (J.L.); zlishaolong@163.com (S.L.); tiantan2355@163.com (T.T.); 2 Intelligent Manufacturing Institute of Laser and Optoelectronic, Wenzhou University, Wenzhou 325035, China; yucao@wzu.edu.cn

**Keywords:** CFRP, low-energy bird strike, barely visible impact damage, multiscale characterization, delamination, compression after impact, AHP–CRITIC evaluation

## Abstract

**Highlights:**

**What are the main findings?**

- A multiscale characterization strategy combining ultrasonic C-scan, 3D profilometry, and SEM effectively revealed the evolution of low-energy bird-strike damage in CFRP laminates.
- Within the impact energy range of approximately 12–33 J, the indentation depth (*P_D_*), delamination area (*S_Da_*), and dissipated energy increased markedly, whereas the compression-after-impact (CAI) strength decreased from 202.2 MPa to 118.9 MPa.
- The dominant damage mode changed with increasing impact energy: internal delamination dominated at relatively lower energy levels, while surface indentation, crack propagation, and intralaminar fracture became more prominent at higher energy levels.
- CAI strength was significantly negatively correlated with both *P_D_* and *S_Da_*, and its variation was more sensitive to *S_Da_*, indicating that internal delamination plays a more critical role in residual compressive strength degradation.
- Based on AHP–CRITIC combined weighting, a quantitative grading framework was established to classify BVID into slight, moderate, severe, and extensive levels.

**What are the implications of the main findings?**

- A single damage metric cannot adequately characterize the complex damage state of CFRP laminates under low-energy bird-strike loading; a multiscale evaluation framework is therefore necessary.
- Because CAI degradation is more sensitive to *S_Da_* than to *P_D_*, internal delamination should be prioritized in residual-strength assessment and maintenance inspections.
- The proposed AHP–CRITIC framework provides a practical route for quantitative BVID grading and supports rapid screening and maintenance decision-making for general aviation composite structures.
- The study offers a methodological basis for extending composite impact assessment from qualitative description to an integrated multiscale quantitative evaluation.

**Abstract:**

Carbon fiber-reinforced polymer (CFRP) laminates are susceptible to barely visible impact damage (BVID) under low-energy bird-strike-like conditions. However, in previous studies, most damage evaluations for BVID were limited to a single scale. In this work, a multiscale characterization and evaluation method integrating the analytic hierarchy process (AHP) and the CRITIC weighting method was proposed to investigate the damage evolution of CFRP laminates under low-energy impacts (approximately 12–33 J). Delamination area (*S_Da_*), indentation depth (*P_D_*), surface crack aspect ratio (*R_A_*), energy dissipation, and compression-after-impact (CAI) strength were analyzed based on phased-array ultrasonic C-scanning, 3D optical profilometry, and scanning electron microscopy. The results showed that *P_D_*, *S_Da_*, and energy dissipation increased from 108.73 μm to 213.93 μm, from 228.6 mm^2^ to 695.8 mm^2^, and from 5.96 J to 21.40 J, respectively, with increasing impact energy. Meanwhile, CAI strength decreased from 202.2 MPa to 118.9 MPa, with a maximum degradation rate of 41.16%. A critical transition was observed in the medium-to-high energy range, where delamination growth gradually plateaued, while intralaminar cracking and fiber fracture became increasingly dominant. The proposed framework enables quantitative grading of BVID severity and provides a practical basis for assessing residual damage in impacted CFRP laminates.

## 1. Introduction

Carbon fiber-reinforced polymer (CFRP), owing to its high specific strength and specific modulus, has been widely used in primary load-bearing aircraft structures, such as wing surfaces [1]. During takeoff and landing, the upper surface of a wing is vulnerable to impacts from small birds. Taking the Cirrus SR20 as a representative example, the flight speed during these phases is typically about 30.8–51.4 m/s [2]. For small birds such as swallows and larks, with a typical body mass of approximately 25 g, the resulting impact energy is approximately 12–33 J, which is consistent with the low-energy impact range defined in ASTM D7136M-12 [3] However, due to the inherent structural characteristics of CFRP, low-energy impacts under small-bird strike-like conditions can readily induce barely visible impact damage (BVID), including matrix cracking, fiber fracture, and delamination [2,4]. Such damage may cause significant degradation of mechanical properties, reduce the likelihood of detection by visual inspection, and consequently increase maintenance costs. The influence of BVID on composite airframe structures has therefore become an important concern in structural design and maintenance. Accordingly, it is particularly important to investigate the evolution and failure characteristics of impact damage in composite materials and to employ multiscale characterization methods to accurately identify the damage features of impacted specimens.

Previous studies have combined experimental and numerical approaches to show that factors such as impact energy [5,6], impact location [7,8], impactor shape [9], material system, and stacking sequence [10,11] are closely associated with the impact damage characteristics of CFRP, leading to many valuable findings. Tuo et al. [12] employed ultrasonic C-scanning, digital image correlation (DIC), scanning electron microscopy (SEM), and a three-dimensional impact damage model based on continuum damage mechanics to characterize the initiation and evolution of internal damage, and systematically evaluated the failure mechanisms of composite laminates under frontal impact. Abdallah et al. [13] carried out low-velocity impact (LVI) and quasi-static indentation (QSI) tests on UD/WF hybrid laminates and, through microscopic observation of the damage, revealed the role of the blocking mechanism in the formation of permanent indentation. Wang et al. [14] used an incremental loading method combined with ultrasonic C-scanning to observe the stress release process in laminates, thereby investigating the buckling behavior and damage mechanisms of CFRP thin plates.

Damage assessment criteria, including indentation depth, delamination area, energy absorption, and compression-after-impact (CAI) strength, are essential for evaluating the damage state of laminated composites. Evci et al. [15] and Li et al. [16] reported that the energy absorption of laminates is closely related to the damage state, whereas its correlation with delamination area is less significant [17,18]. In addition, the indentation depth on the impacted surface has been shown to be positively correlated with the impact energy [19,20]. Zhang et al. [21] investigated the relationships among impact energy, damage area, and CAI strength by combining experimental and numerical approaches. Their results indicated that once the impact energy exceeded a threshold, further increases did not lead to substantial changes in either the damage area or the CAI strength. Similarly, some researchers have also used the crack length on the impacted surface [22,23] to evaluate the damage state. However, current studies have still mainly focused on high-velocity, high-energy impacts, while insufficient attention has been paid to low-velocity, low-energy impacts. Moreover, reliance on a single damage assessment criterion cannot provide an accurate characterization of the damage state of laminates.

To address the above issues, drop-weight impact tests were conducted to investigate the effects of impact energy on the damage characteristics and evolution mechanisms of CFRP laminates. A macro–meso–micro multiscale framework was established to analyze the impact damage morphology and mechanical response of the laminates. Within this framework, the indentation depth (*P_D_*) and surface crack aspect ratio (*R_A_*) were selected as evaluation criteria for surface damage characteristics, while the delamination area (*S_Da_*) was used as the evaluation criterion for internal damage. On this basis, the low-energy impact damage mechanisms of CFRP laminates were further analyzed, and the correlations among these evaluation criteria were discussed in conjunction with energy dissipation, thereby providing data support and a theoretical reference for improving the evaluation index system of general aviation aircraft.

## 2. Materials and Methods

### 2.1. Fabrication of CFRP Laminates and Specimens

In this study, CFRP laminates were fabricated from a medium-temperature plain-woven T300 3K/2019B carbon fiber/epoxy composite prepreg, with a resin volume content of 40%, using an autoclave molding process. The prepreg stacking sequence of each laminate was [0]28, and the nominal thickness of a single prepreg ply was 0.2 mm. The laminates were subsequently cut into specimens measuring 150 mm × 100 mm using a precision cutting machine for composite materials. The average thickness of the sample is 5.15 mm.

### 2.2. Compression-After-Impact (CAI) Test

**Figure 1:**
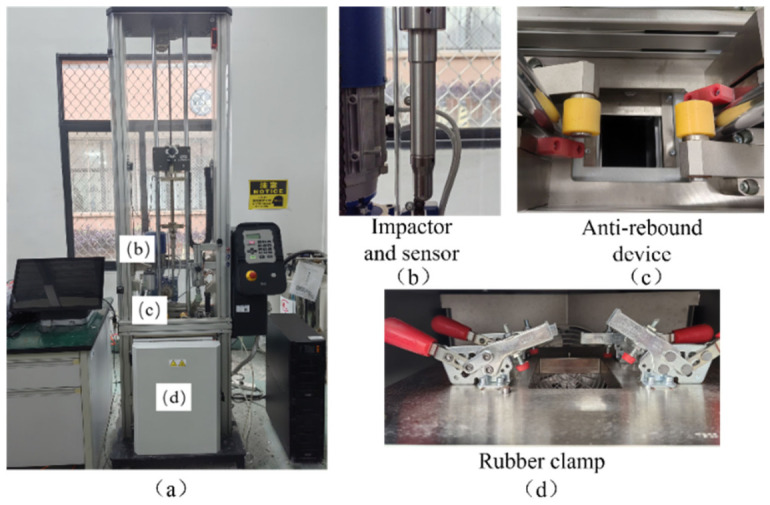
Drop-weight impact test system. (**a**) Drop weight impact testing machine; (**b**) Impactor and sensor; (**c**) Anti-rebound device; (**d**) Rubber clamp.

According to ASTM D7136M-12, drop-weight impact tests were conducted using an Instron 9440 impact testing machine. A hemispherical impactor with a diameter of 12.7 mm and a total mass of 3.265 kg was used, as shown in Figure 1. The specimen was fixed on a flat support by four rubber clamps in the standard fixture. During the tests, the impactor was released from rest with an initial velocity of 0 m/s, and the target impact energy was controlled by adjusting the drop height according to the relationship *E* = *mgh*. The corresponding drop heights were calculated for different impact energy levels (T1–T5). In addition, the testing system was equipped with an anti-rebound device, which was turned on during the tests to prevent secondary impacts from influencing the damage results. No additional clamp force was independently controlled during the tests. The parameters of the drop-weight impact tests are listed in Table 1.

**Figure 2:**
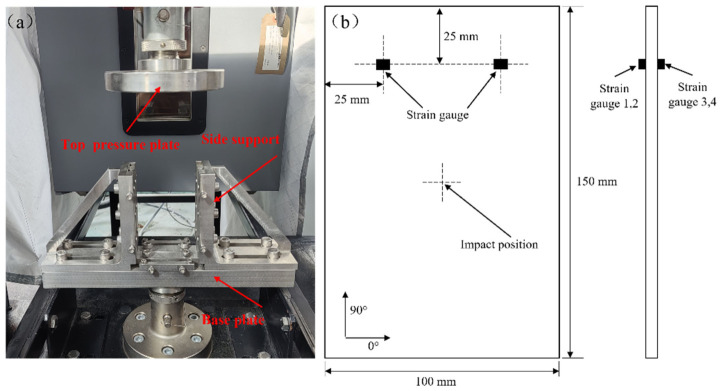
Compression-after-impact (CAI) test: (**a**) test procedure; (**b**) test specimen.

Compression-after-impact (CAI) tests of the composite specimens were conducted on an Instron 5982 universal testing machine in accordance with ASTM D7137 [24]. The fixture used for the CAI tests is shown in Figure 2. The bottom side of the specimen was secured by a base plate, while lateral support elements restrained both sides of the specimen to prevent buckling.

### 2.3. Multiscale Characterization of Impact Damage

In the correlation analysis of damage assessment criteria, five indicators, namely the indentation depth (*P_D_*), surface crack aspect ratio (*R_A_*), delamination area (*S_Da_*), energy dissipation, and compression-after-impact (CAI) strength, were adopted to evaluate the post-impact damage characteristics of the laminates.

**Figure 3:**
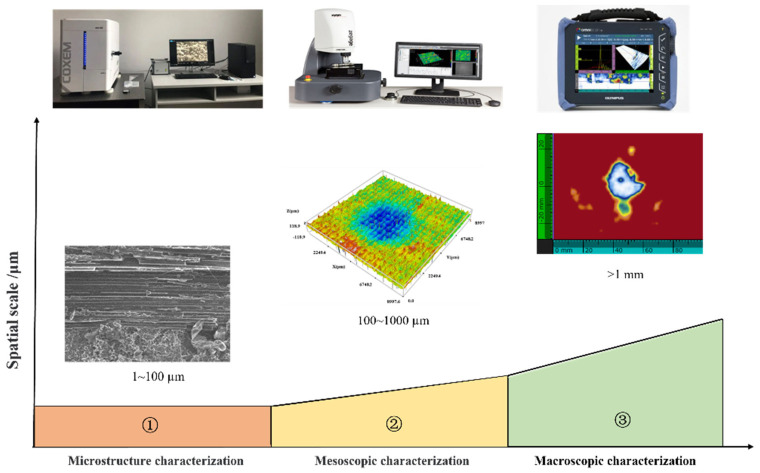
Multiscale characterization of low-energy impact damage in CFRP.

After the impact tests, the impact-induced damage was characterized using a multiscale approach, as shown in Figure 3. The intralaminar damage morphology of the laminates was characterized at the microscale using a tabletop scanning electron microscope (SS-60S-ST). Through the interaction between the electron beam and the material, various physical signals were generated, collected, amplified, and re-imaged to characterize the microstructural morphology of the material, thereby revealing microscopic features such as fiber fracture and matrix cracking within the laminate. The mesoscale observation of the post-impact surface morphology was carried out using a 3D optical surface profilometer (Sneox090). Using the field-of-view stitching function, with a magnification of 50× and a resolution of 5 mm, the original coordinates of each point on the surface morphology were acquired. Based on these data, the indentation depth (*P_D_*) and surface crack aspect ratio (*R_A_*) of each specimen were calculated within 10 min after impact. Furthermore, phased-array ultrasonic C-scanning (OmniSX-PA1664PR) was employed for macroscale characterization of the interlaminar damage morphology of the laminates. During inspection, linear scanning was performed in pulse-echo mode, and different delay laws were applied to the individual elements of the probe to control the beam. In this way, the delamination morphology was obtained, from which the delamination area (*S_Da_*) was subsequently calculated.

### 2.4. Correlation Analysis and Comprehensive Evaluation Method

#### 2.4.1. Selection of Evaluation Indicators

The statistical results of multi-scale damage indicators under different impact energy levels are shown in Table 2.

#### 2.4.2. AHP–CRITIC-Based Comprehensive Evaluation Method

Combined with previous findings reported in the literature, it can be concluded that the indentation depth *P_D_* and delamination area *S_Da_* more directly reflect the damage state of CFRP laminates and can therefore be regarded as the two most important evaluation criteria. The importance of the surface crack aspect ratio *R_A_* is slightly lower than that of these two criteria, whereas energy dissipation *E_Dissipation_* and CAI strength are of relatively lower importance among the five evaluation criteria. On this basis, a pairwise comparison matrix was adopted to determine the relative importance of each criterion. To compare each pair of criteria, corresponding scores were assigned according to their relative importance in damage evaluation. The scoring system typically uses values from 1 to 9: 1 indicates that the two criteria are equally important; 3 indicates that one criterion is slightly more important than the other; 5 indicates that one criterion is obviously more important than the other; 7 indicates that one criterion is much more important than the other; and 9 indicates that one criterion is extremely more important than the other. The scores assigned to the different damage evaluation criteria are presented in Table 3.

Based on the scoring results shown in Table 3, the weights of the evaluation criteria were determined using the eigenvector method. The maximum eigenvalue of the pairwise comparison matrix and its corresponding eigenvector were first calculated. The eigenvector was then normalized such that the sum of all its elements was equal to 1, and the normalized values were taken as the weights of the respective criteria. The obtained weights are listed in Table 4.

To ensure that the judgment logic in the matrix is consistent with the actual situation, a consistency test can be performed on the pairwise comparison matrix of the damage assessment criteria. For the above pairwise comparison matrix, the maximum eigenvalue is $\lambda_{max}$ = 5.2669, and the consistency index (CI) can be calculated using Equation (1):

(1) $$CI=\frac{\lambda_{max}-n}{n-1}$$

Here, *n* denotes the order of the pairwise comparison matrix (*n* = 5 in this study). The consistency ratio (CR) can be calculated using Equation (2):

(2) $$CR=\frac{CI}{RI}$$

Here, RI is the random consistency index, which is determined based on the eigenvalues of randomly generated matrices. For *n* = 5, RI = 1.12 can be obtained from the standard table of the AHP method. According to the above equations, CI = 0.0667 and CR = 0.0596 can be calculated. Since CR < 0.1, the matrix is considered to have good internal consistency, indicating that the weight allocation of the damage assessment criteria is reasonable.

In the preceding sections, a multiscale indicator system was established based on the analytic hierarchy process (AHP), and the weights of the corresponding indicators were determined. AHP can reflect the hierarchical structure of the indicators and incorporate expert knowledge; however, because its weights are essentially derived from the judgment matrix, a certain degree of subjectivity remains unavoidable. To reduce the bias associated with relying on a single subjective weighting method, and to ensure that the weights can reflect both the discriminative ability of different indicators across samples and the complementarity of information among indicators, the CRITIC (Criteria Importance Through Intercriteria Correlation) objective weighting method was further introduced and combined with the AHP-derived weights. This yielded a multiscale integrated weighting system that accounts for both empirical knowledge and data characteristics, thereby improving the objectivity and robustness of the comprehensive evaluation results.

Assume that there are m samples, which may represent either individual specimens or statistical values at different impact energy levels, and n evaluation indicators, with the original data matrix denoted as X = [*x_ij_*]. Since the indicators differ in both dimensional units and evaluation orientation, directional unification and normalization are first required. If damage severity is taken as the evaluation objective, delamination area *S_Da_*, indentation depth *P_D_*, crack aspect ratio *R_A_*, and energy dissipation *E_Dissipation_* are regarded as cost-type indicators, whereas residual compressive strength CAI is regarded as a benefit-type indicator. In this study, range normalization was adopted to obtain the dimensionless matrix Z = [$z_{ij}$].

Benefit-type indicators:

$$z_{ij}=\frac{x_{ij}-\min\left(x_{j}\right)}{\max\left(x_{j}\right)-\min\left(x_{j}\right)}$$

Cost-type indicators:

$$z_{ij}=\frac{max(x_{j})-x_{ij}}{\max\left(x_{j}\right)-min(x_{j})}$$

After the above processing, all indicators were transformed to satisfy a unified orientation in which a larger value indicates a better state (i.e., less severe damage), thereby laying the foundation for subsequent weight calculation and comprehensive evaluation.

The CRITIC method assumes that the importance of an indicator is jointly determined by two aspects: its ability to discriminate among samples (contrast intensity) and its complementarity with other indicators (conflict or redundancy reduction). Based on the normalized matrix Z, the standard deviation *σ_j_* of the *j*th indicator is calculated to characterize its degree of dispersion, namely its discriminative ability. Meanwhile, the correlation coefficient, $r_{jk}$, between indicators *j* and *k* is calculated to reflect the degree of information overlap. The information content of indicator *j* is then defined as follows:

$$C_{j}=\sigma_{j}\sum_{k=1}^{n}\left(1-r_{jk}\right)$$

In this expression, $\sum_{k=1}^{n}\left(1-r_{jk}\right)$ describes the complementarity between a given indicator and the remaining indicators. When an indicator is highly correlated with other indicators, $r_{jk}$ becomes relatively large and $\left(1-r_{jk}\right)$ correspondingly becomes small, indicating substantial information overlap and high redundancy with the other indicators; consequently, its overall information content is weakened. Conversely, the indicator provides a more independent information contribution. Finally, the objective weight in the CRITIC method is defined as follows:

$$w_{j}^{CRI}=\frac{C_{j}}{\sum_{j=1}^{n}C_{j}},j=1,2,\ldots ,n$$

The calculated weights are listed in Table 5 below.

To simultaneously preserve the capability of AHP to reflect the structural hierarchy of the indicator system and engineering experience, while introducing the objective constraints of CRITIC in terms of indicator discriminative ability and complementarity, a combined weighting strategy was adopted to integrate the two types of weights. Let the AHP-derived weight be denoted by $w_{j}^{AHP}$ and the objective CRITIC weight by $w_{j}^{CRI}$ The combined weight is then defined as a linear combination:

$$w_{j}^{*}=\alpha w_{j}^{AHP}+\left(1-\alpha \right)w_{j}^{CRI},\alpha [0,1]$$

Here, α is the combination coefficient used to adjust the relative contributions of subjective and objective information. When α = 0.5, the two are integrated with equal weights; when α takes a larger value, greater emphasis is placed on engineering experience; when α takes a smaller value, greater emphasis is placed on data objectivity. To ensure that the combined weights satisfy the normalization requirement, $w_{j}^{*}$ can be further normalized so that $\sum_{j=1}^{n}w_{j}^{*}=1$. In this study, α was set to 0.5.

By substituting the combined weights $w_{j}^{*}$ into the comprehensive evaluation model, the overall score of each sample can be obtained as follows:

$$S_{i}=\sum_{j=1}^{n}w_{j}^{*}z_{ij},i=1,2,\ldots ,m$$

**Figure 4:**
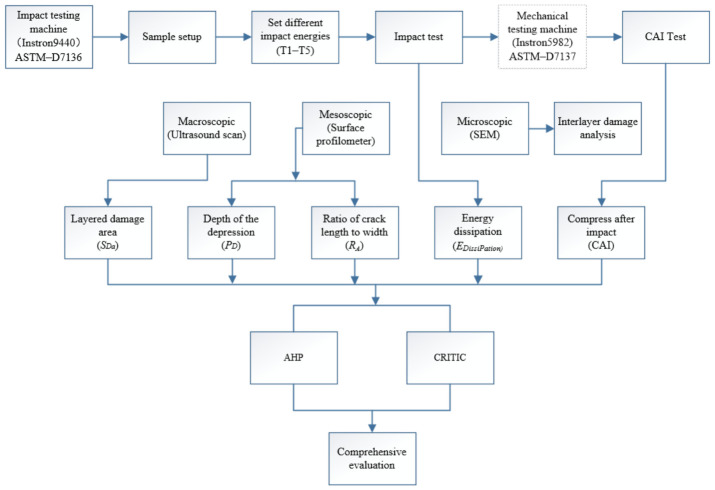
Research progress flowchart.

Here, $S_{i}$ denotes the comprehensive evaluation value of the *i*th sample. Based on the magnitude of $S_{i}$, different impact energy levels can be ranked. Moreover, BVID grades can be classified by combining quantile thresholds, the natural breaks method, or engineering experience-based thresholds. This weighting system simultaneously reflects the trade-off among macroscale-related (delamination), mesoscale-related (geometric morphology), and microscale-related (mechanism identification) information in the overall evaluation. Therefore, it can more fully capture the complementary relationships among multiscale indicators and avoid the evaluation bias that may arise from relying on a single-scale indicator alone. The research progress of this paper is shown in Figure 4.

## 3. Results

### 3.1. Mechanical Response Under Different Impact Energies

**Figure 5:**
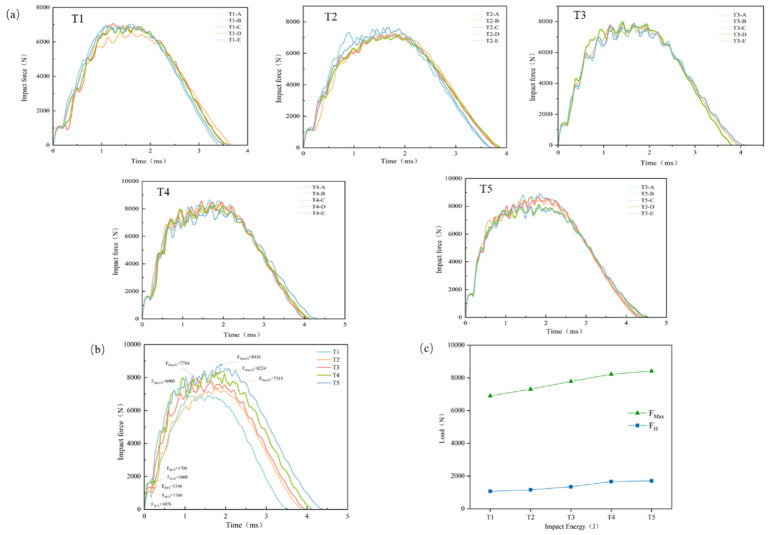
Impact force–time curves of CFRP laminates: (**a**) curves under five impact energy levels; (**b**) curves of typical specimens; (**c**) comparison of different impact energies (*F_H_* and *F_M__ax_*).

The delamination failure of composites can be characterized by the Hertzian failure load *F_H_* [25]. Owing to the different levels of potential energy stored in the impactor, the impact force rises to the maximum impact load *F_Max_* following different loading trends. The maximum impact load *F_Max_* is governed by the material properties of the composite laminate and influences damage modes dominated primarily by fiber fracture. To investigate the relationship between impact force and internal damage in CFRP laminates, the fast Fourier transform (FFT) method was used to remove impact oscillations from the experimental signals, and the resulting impact force–time curves under different impact energies are shown in Figure 5a. Comparison of the curves shows that all groups exhibit consistent variation trends, and the coefficients of variation of both *F_H_* and *F_Max_* are less than 10%, satisfying engineering requirements and confirming the reliability of the present experiments. As shown in Stage 1 of Figure 5a, the impact force increases linearly before the curve reaches the first peak. In Stage 2, after the impact force reaches *F_H_*, the curve gradually tends to level off. In Stage 3, when the energy input ceases, the curve decreases gradually.

Figure 5b,c show the impact force–displacement curves of typical specimens. The growth rates of *F_H_* from specimen groups T1 to T5 were 8.41%, 16.03%, 23.32%, and 2.89%, respectively, indicating that the resistance of the laminate to initial delamination increased with increasing impact energy, and reached its maximum in the T3–T4 interval. The growth rates of *F_Max_* were 5.59%, 6.44%, 5.65%, and 2.33%, respectively, indicating that the maximum impact resistance of the laminate also increased with increasing impact energy and became optimal in the T2–T3 interval. However, when the impact energy reached T5, the growth rates of both *F_H_* and *F_Max_* decreased markedly to 2.89% and 2.33%, respectively, compared with the preceding groups. This may be because the specimen had already reached its maximum resistance to delamination damage at the T4 energy level.

**Figure 6:**
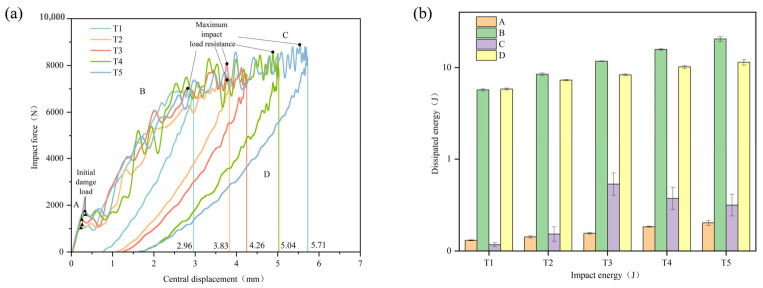
(**a**) Impact force–central displacement curves of typical specimens at different impact energies.; (**b**) Typical energy absorption in different regions under different impact energies.

The impact force–central displacement curves of typical specimens are presented in Figure 6. As shown in Figure 6, the curves can be divided into four characteristic regions corresponding to damage initiation, damage propagation, damage oscillation, and rebound. A distinct inflection point was observed after the maximum load, suggesting that it may serve as a characteristic feature for identifying changes in the damage state of the laminates.

### 3.2. Multiscale Characterization Results

The statistical results of the multiscale damage indicators are summarized in Table 2. As listed in Table 2, *P_D_* increased from 108.73 μm at T1 to 213.93 μm at T5, *S_Da_* increased from 228.6 mm^2^ to 695.8 mm^2^, and dissipated energy increased from 5.96 J to 21.40 J. In contrast, *R_A_* remained relatively stable from T1 to T4 and increased markedly at T5.

**Figure 7:**
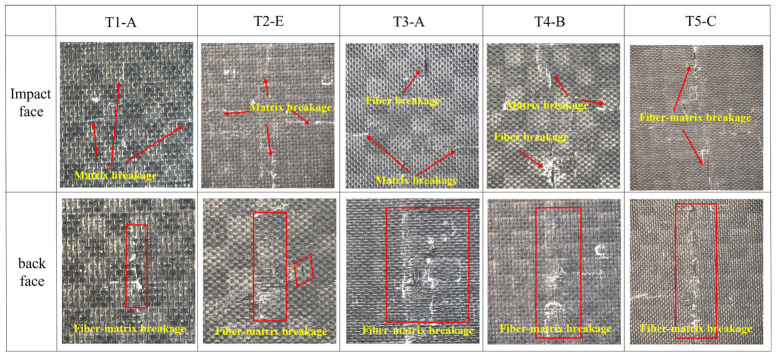
Surface impact damage modes of typical specimens.

The surface impact damage modes of typical specimens are shown in Figure 7. It can be observed that the front-surface damage gradually intensified with increasing impact energy. At T1 and T2, matrix cracking was the dominant failure mode, whereas fiber pull-out and fiber fracture became apparent at T3–T5. On the rear surface, penetrating damage was already observed at T1, and obvious delamination and matrix spalling appeared at T4.

**Figure 8:**
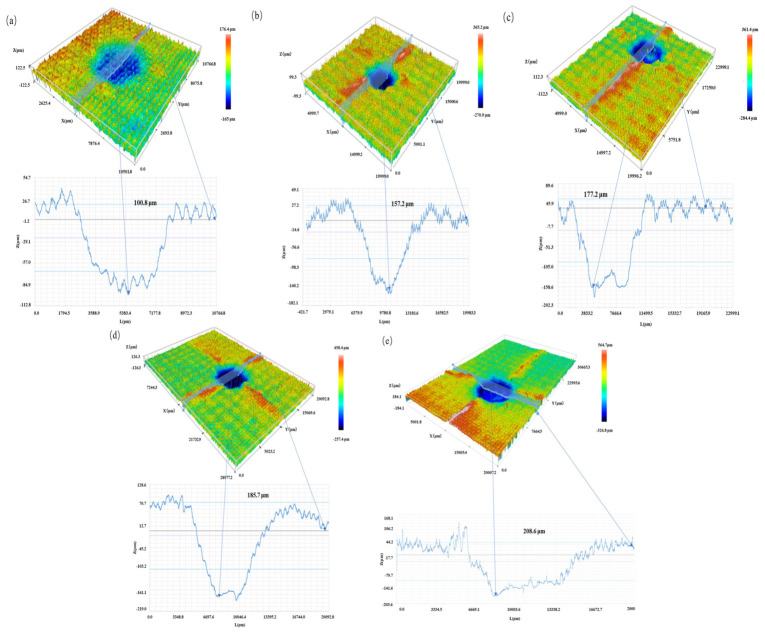
Front-surface impact damage morphology of typical specimens. (**a**) T1; (**b**) T2; (**c**) T3; (**d**) T4; (**e**) T5.

The front-surface impact damage morphology obtained by 3D profilometry is shown in Figure 8. As shown in Figure 8, the permanent indentation became deeper, and the surface crack morphology became more pronounced with increasing impact energy, consistent with the variation trend of *P_D_* and *R_A_* listed in Table 2.

**Figure 9:**
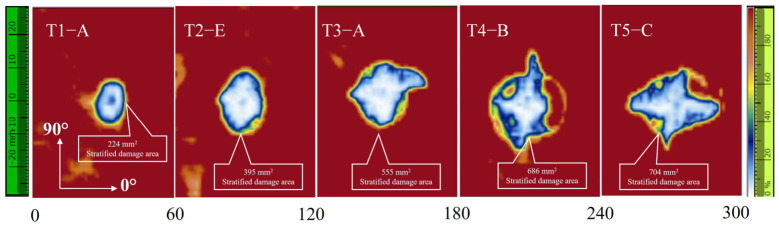
Ultrasonic C-scan images of delamination damage in typical specimens.

The internal delamination damage characterized by ultrasonic C-scan is shown in Figure 9. As shown in Figure 9, the internal damage morphology evolved from approximately elliptical regions at lower energies to more irregular damage patterns at higher energies. The delamination area increased nearly linearly in the lower energy range and then tended to level off after T4.

**Figure 10:**
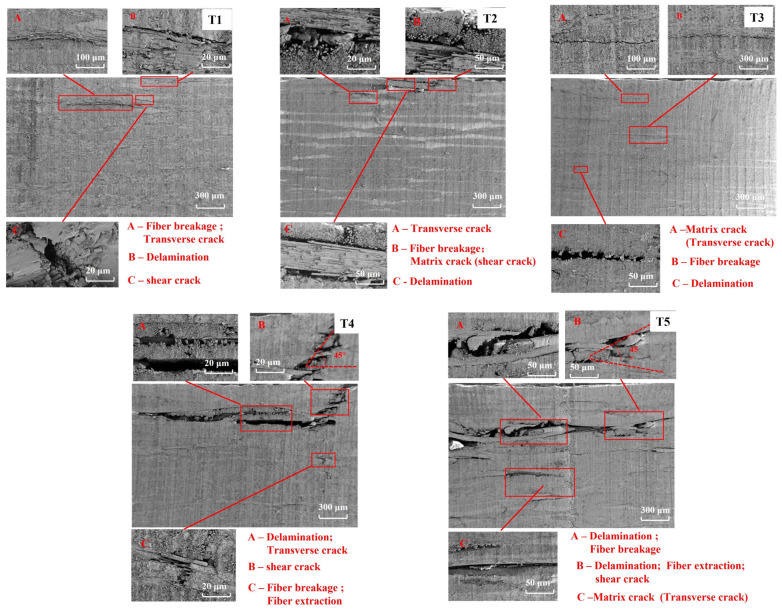
SEM characterization of the internal damage microstructure of CFRP laminates.

The SEM characterization results are shown in Figure 10. As shown in Figure 10, the internal microscopic damage evolved progressively with increasing impact energy, involving matrix cracking, fiber debonding, and fiber fracture. These observations provide microscopic evidence for the macroscopic and mesoscopic damage features described above.

### 3.3. Compression-After-Impact Strength

**Figure 11:**
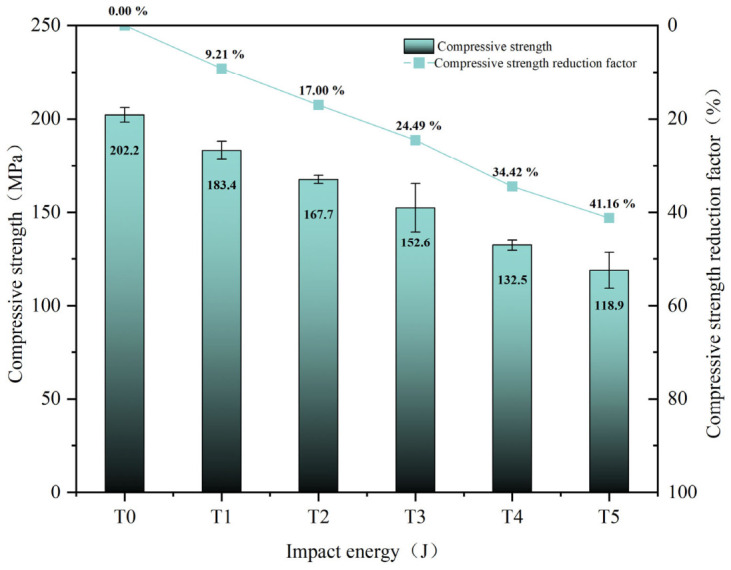
CAI strength and strength degradation rate.

The CAI strength and the corresponding strength degradation rate are shown in Figure 11, respectively, where T0 denotes the unimpacted specimen. As shown in Figure 11, the reduction in CAI strength became more pronounced with increasing impact energy, decreasing from 202.2 MPa for T0 to 118.9 MPa for T5, with a maximum degradation rate of 41.16%. These results indicate that even low-energy impact damage can significantly impair the residual compressive load-bearing capacity of CFRP laminates.

### 3.4. Correlation Analysis and Evaluation Results

#### 3.4.1. Correlation Analysis Among Damage Indicators

**Figure 12:**
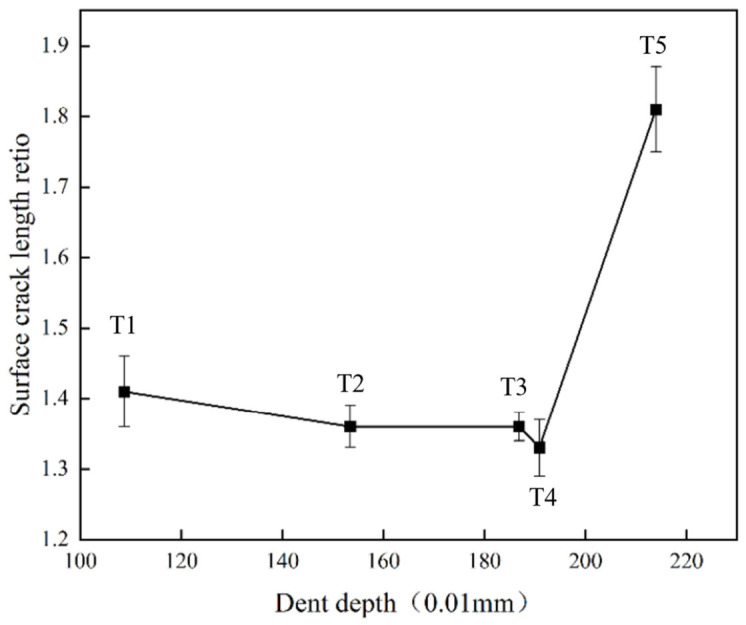
Correlation between *P_D_* and *R_A_*.

Based on all the experimental results listed in Table 2 and Figure 11, the correlations among the damage assessment criteria can be summarized. The relationship between the indentation depth *P_D_* and the surface crack aspect ratio *R_A_* is shown in Figure 12. With increasing impact energy, the evolution of *P_D_* and *R_A_* exhibits a segmented trend, characterized by an initial stage of relative stability followed by a marked increase.

**Figure 13:**
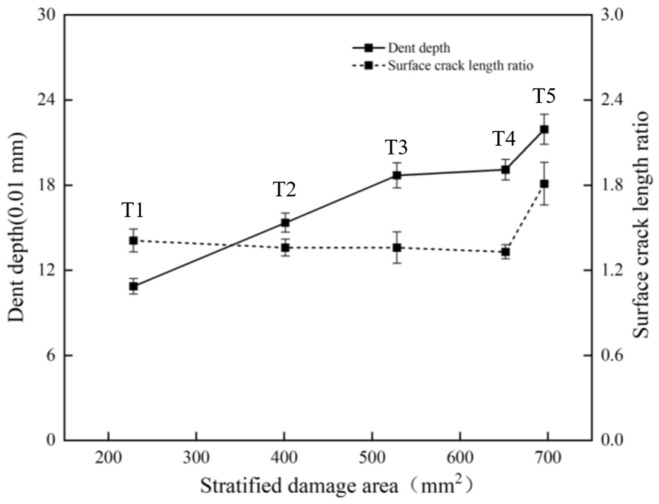
Correlation between *P_D_* and *S_Da_*.

The relationship between *P_D_* and *S_Da_* is shown in Figure 13. As shown in Figure 13, both indicators increased with impact energy, but their growth rates differed with damage stage, indicating that the balance between surface intralaminar damage and internal interlaminar damage changed during impact evolution.

**Figure 14:**
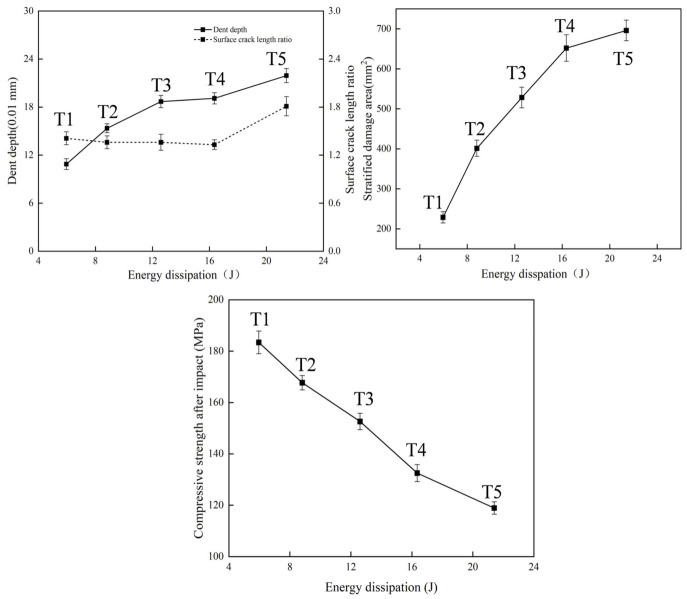
Correlations among *P_D_*, *S_Da_*, *R_A_*, CAI, and *E_Dissipation_*.

The correlations among *P_D_*, *S_Da_*, *R_A_*, CAI, and dissipated energy are further illustrated in Figure 14. As shown in Figure 14, both *P_D_* and *S_Da_* increased monotonically with increasing dissipated energy, whereas CAI strength decreased continuously, showing a significant negative correlation with impact-induced damage severity.

**Figure 15:**
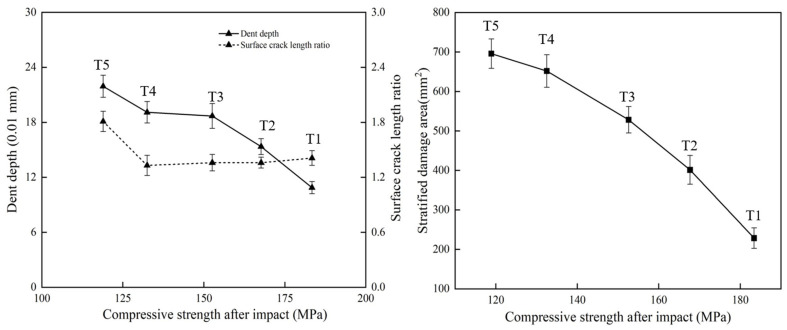
Correlations between CAI strength and *P_D_*, *S_Da_*, and *R_A_*.

The relationships between CAI strength and *P_D_*, *S_Da_*, and *R_A_* are shown in Figure 15. As shown in Figure 15, CAI strength was significantly negatively correlated with both *P_D_* and *S_Da_*, whereas no clear monotonic relationship was observed between CAI strength and *R_A_*. Moreover, the variation trend of *S_Da_* was more consistent with the degradation of CAI strength than that of *P_D_*, indicating that residual compressive strength is more sensitive to internal delamination damage.

#### 3.4.2. Comprehensive Evaluation and BVID Grading Result

Using the above equations, the comprehensive scores for different impact energy levels were calculated, as presented in Table 6.

Specifically, damage with a comprehensive score ≤ 0.5 is classified as slight BVID, indicating a low degree of damage in the laminate at the corresponding impact energy, typically manifested by very small surface cracks, slight indentations, or matrix microcracks, with negligible influence on the overall material performance. Damage with a comprehensive score between 0.5 and 0.8 is classified as moderate BVID, indicating the presence of slightly deeper indentations, longer cracks, or slight local interlaminar delamination, which exerts only a limited influence on material performance. Damage with a comprehensive score between 0.8 and 1.0 is classified as severe BVID, indicating expanded interlaminar delamination, longer crack propagation, or local fiber fracture, which results in a noticeable degradation of material performance. Damage with a comprehensive score ≥ 1.0 is classified as extensive BVID, indicating large-area interlaminar delamination, intralaminar crack propagation, and fiber fracture, thereby causing a relatively significant deterioration in performance.

## 4. Discussion

### 4.1. Damage Evolution Mechanism

**Figure 16:**
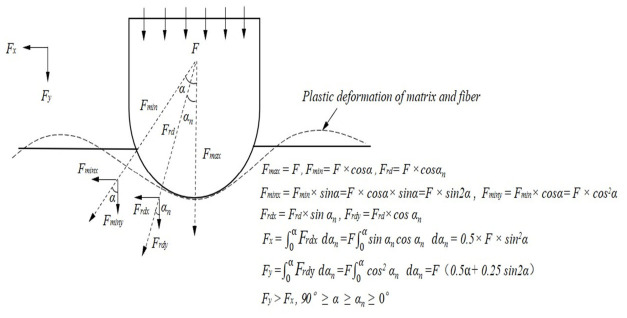
Global damage behavior of CFRP specimens under impact.

The global damage behavior of CFRP specimens under impact is schematically illustrated in Figure 16. The impact damage evolution of CFRP laminates under low-energy loading is governed by the partition of impact energy into global deformation and local failure. At relatively low energy levels, matrix cracking and interlaminar delamination dominate because the matrix reaches its failure limit earlier than the fibers. As the impact energy increases, tensile and bending stresses become more severe, promoting fiber fracture, crack propagation, and local surface geometric damage. This explains why *S_Da_* grows rapidly in the lower energy range, whereas *P_D_* and *R_A_* become more pronounced at higher energies.

**Figure 17:**
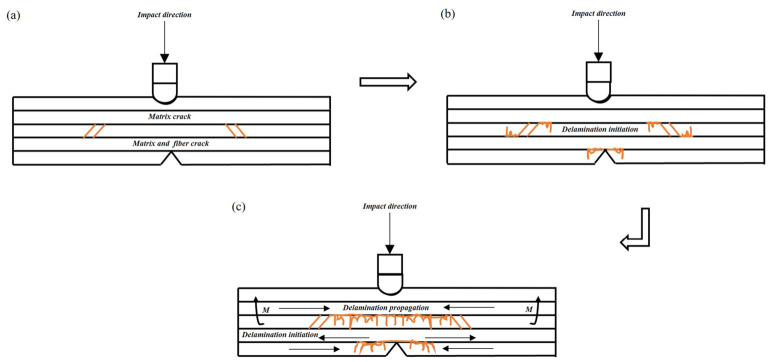
Schematic illustration of the delamination damage mechanism in CFRP specimens. (**a**) Crack initiation; (**b**) Layer initiation; (**c**) Layer propagation.

The delamination damage mechanism is further illustrated in Figure 17. There are two main causes of delamination. On the one hand, relative sliding between adjacent plies generates interlaminar shear stress, which in turn induces interlaminar damage. On the other hand, stress-wave reflection at the rear surface generates bending stresses, which accelerate the initiation and propagation of delamination. As shown in Figure 17a–c, intralaminar cracks propagate toward interlaminar regions, resulting in delamination between adjacent plies. When the impact energy increases further, the fibers fracture after exceeding their strength limit, and the specimen reaches its maximum resistance to delamination damage, after which the delamination damage no longer continues to propagate.

### 4.2. Relationship Between Multiscale Damage and Residual Strength

The stronger sensitivity of CAI strength to *S_Da_* than to *P_D_* indicates that internal delamination plays a more critical role in residual compressive failure than visible surface indentation. This is mechanically reasonable because delamination reduces interply load transfer capability and promotes local instability during compression loading. Consequently, even when surface damage appears limited, extensive internal delamination may still lead to severe strength degradation. As indicated by Figure 16, *S_Da_* is therefore a more representative indicator for assessing residual compressive strength degradation.

Although CAI strength was found to be more sensitive to *S_Da_* than to *P_D_*, the weighting framework was established for comprehensive damage evaluation rather than for residual strength prediction alone. Therefore, *P_D_* and *S_Da_* were assigned equal weights in AHP because they represent two dominant but complementary aspects of damage severity, namely surface damage and internal delamination.

This finding also highlights the importance of integrating both internal and external damage descriptors in damage evaluation. Surface features such as *P_D_* and *R_A_* are useful for rapid morphological assessment, but *S_Da_* is more directly associated with structural performance loss. Accordingly, internal damage indicators should receive higher priority in the engineering assessment of BVID.

### 4.3. Significance of the AHP–CRITIC Evaluation Framework

The proposed AHP–CRITIC framework provides a practical route for integrating multiscale indicators into a single quantitative evaluation system. Compared with using a single damage parameter, the combined method better accounts for the complementary information contained in surface morphology, internal delamination, dissipated energy, and residual strength. The integration of AHP and CRITIC also improves the balance between engineering judgment and data-driven weighting, thereby enhancing the interpretability and robustness of the evaluation results. The final grading results listed in Table 6 further demonstrate that the proposed framework can effectively classify BVID severity under different impact energy levels.

It should be noted that the laminate configuration used in this study was a unidirectional [0]28 lay-up, which was selected as a simplified model to facilitate damage mechanism interpretation and evaluation framework development. Therefore, caution is required when directly extending the present conclusions to quasi-isotropic or more complex aerospace laminate configurations.

## 5. Conclusions

In this study, the analytic hierarchy process (AHP) and the CRITIC objective weighting method were combined to assign weights to the damage assessment criteria. The weights of the indicators were determined using the eigenvector method, and the rationality of the evaluation criteria was verified through the construction of the pairwise comparison matrix and the consistency test. Furthermore, comprehensive scores under different impact energy levels were calculated using the weighted average method. Based on these scores, the damage was classified into four grades, namely slight BVID, moderate BVID, severe BVID, and extensive BVID. On this basis, a multiscale characterization-based evaluation method for low-energy impact damage in CFRP was established. The main conclusions are as follows.

- Within the impact energy range of approximately 12–33 J, both damage severity and energy dissipation increased significantly with increasing impact energy. As listed in Table 2, *P_D_* increased from 108.73 μm to 213.93 μm, *S_Da_* increased from 228.6 mm^2^ to 695.8 mm^2^, and *E_Dissipation_* increased from 5.96 J to 21.40 J. These results indicate that even low-energy impact can induce pronounced evolution of both internal and surface damage.
- Impact damage caused a significant degradation in the residual load-bearing capacity of the structure. As shown in Figure 11, the CAI strength decreased from 202.2 MPa in the unimpacted state T0 to 118.9 MPa in T5, corresponding to a maximum degradation rate of 41.16%. Correlation analysis further showed that CAI was significantly negatively correlated with both *P_D_* and *S_Da_*, and that CAI was more sensitive to *S_Da_*, indicating that the delamination area *S_Da_* can serve as a key indicator for characterizing CAI degradation.
- By combining AHP and CRITIC weighting methods, a comprehensive evaluation model was established. As listed in Table 5, the damage states under different impact energy levels were quantitatively classified into four grades: slight BVID, moderate BVID, severe BVID, and extensive BVID. This model provides a practical basis for rapid damage assessment and maintenance decision-making for general aviation composite structures.

## Figures and Tables

**Table 1:** Parameters of the drop-weight impact tests.

Type	Impact Energy	Impact Velocity	Impactor Mass	Number of Test Pieces
T1	11.9 J	2.69 m/s	3.265 kg	5
T2	16.2 J	3.14 m/s		5
T3	21.2 J	3.59 m/s		5
T4	26.8 J	4.04 m/s		5
T5	33.1 J	4.49 m/s		5

**Table 2:** Statistical results of multiscale damage indicators under different impact energy levels.

Type	*P_D_* (μm)	*R_A_*	*S_Da_* (mm^2^)	*E_Disspation_* (J)	*E_Disspation_/E_Impact_* (%)
T1	108.73 ± 7.21	1.41	228.6 ± 14.78	5.96	50.46
T2	153.50 ± 5.73	1.36	401.3 ± 11.96	8.81	54.36
T3	186.91 ± 4.33	1.36	528.2 ± 22.05	12.60	59.42
T4	190.89 ± 5.71	1.33	651.8 ± 9.93	16.35	60.99
T5	213.93 ± 8.98	1.81	695.8 ± 32.41	21.40	64.66

**Table 3:** Pairwise scoring matrix for the damage assessment criteria.

Standard	*P_D_*	*S_Da_*	*R_A_*	*E_Dissipation_*	CAI
*P_D_*	1	1	5	7	7
*S_Da_*	1	1	5	7	7
*R_A_*	1/5	1/5	1	5	5
*E_Dissipation_*	1/7	1/7	1/5	1	1
CAI	1/7	1/7	1/5	1	1

**Table 4:** Weights of the damage assessment criteria.

Standard	*P_D_*	*S_Da_*	*R_A_*	*E_Dissipation_*	CAI
Weight	0.3897	0.3897	0.1359	0.0424	0.0424

**Table 5:** Calculated CRITIC weights.

	Indicator Variability	Indicator Conflict	Information Content	Weight
*P_D_*	0.388	0.610	0.237	14.1061%
*R_A_*	0.419	1.918	0.803	47.8540%
*S_Da_*	0.408	0.603	0.246	14.6557%
*E_Dissipation_*	0.394	0.485	0.191	11.3897%
CAI	0.403	0.500	0.201	11.9945%

**Table 6:** Comprehensive scores for different impact energy levels.

Type	T1	T2	T3	T4	T5
Comprehensive Evaluation score	0.29	0.52	0.72	0.84	1.04

## Data Availability

The original contributions presented in this study are included in the article. Further inquiries can be directed to the corresponding author.
